# Supplementary material for: Are carbon emissions trading and green financial instruments synergistic? -Comprehensive quantitative research based on content analysis
Source: PLoS One. 2024 Mar 7;19(3):e0298601. doi: 10.1371/journal.pone.0298601 (PMC10919601; doi:10.1371/journal.pone.0298601)
Supplement: S1 Data — (ZIP) [file pone.0298601.s001.zip › Data/地级市Z_F工作报告.zip!/╣≤╓▌/╫±╥σ╩╨/└┤╫╘╣1⁄2╓┌║┼í╛┬φ┐╦╩2╛▌═°í┐_2019.docx]

各位代表：

　　现在，我代表市人民政府向大会作工作报告，请予审议，并请各位政协委员和其他列席人员提出意见。

　　2018年政府工作回顾

　　过去一年，我们在省委、省政府和市委的坚强领导下，在市人大、市政协的监督支持下，以习近平新时代中国特色社会主义思想为指导，深入贯彻党的十九大精神和习近平总书记在贵州省代表团重要讲话精神，坚持稳中求进工作总基调，践行新发展理念，落实高质量发展要求，全力推进供给侧结构性改革，坚守发展和生态两条底线，统筹推进稳增长、促改革、调结构、惠民生、防风险各项工作，全市经济社会发展稳中有进、稳中向好。初步统计，完成地区生产总值3000.23亿元，增长10.4%，排全省第3位；规模工业增加值增长12.1%，排全省第1位；固定资产投资增长17.8%，排全省第2位；一般公共预算收入达252.14亿元，增长16.5%，排全省第1位；社会消费品零售总额增长8.7%，排全省第2位；金融机构存贷款余额分别增长3.3%、19.5%；城乡居民人均可支配收入分别达32312元、12265元，分别增长9%、10.2%；全面小康实现程度达92%。一年来，我们坚持发展第一要务，紧紧围绕黔川渝结合部中心城市战略定位，积极推进“一枢纽两中心三基地”建设，综合实力跃上新台阶，经济增速高于全国、高于西部、高于全省。一年来，我们坚决打好“三大攻坚战”，政府债务风险总体可控，没有发生区域性、系统性金融风险，夺取了脱贫攻坚关键之年的决定性胜利，生态环境质量持续改善。一年来，我们尽力而为、量力而行，持续保障和改善民生事业，人民群众获得感、幸福感、安全感持续增强。

　　我们主要抓了以下工作：

　　一、尽锐出战发起总攻，脱贫攻坚再创新佳绩。深入实施大扶贫战略行动，全力打好“四场硬仗”，狠抓“五个专项治理”，脱贫攻坚连战连捷。桐梓、习水、湄潭、凤冈脱贫摘帽，210个贫困村出列，减少贫困人口10.52万人，贫困发生率降到2.02%。贫困户“一长两短”产业及利益联结机制更加优化，贫困人口人均可支配收入增长24.4%。建成“组组通”公路7662公里，实现通组公路全覆盖，完成农村电网改造1.4万公里，农村饮水安全新增受益人口25.8万人。建立“三大体系”后续扶贫机制，实施易地扶贫搬迁2.36万人。教育扶贫力度空前，贫困人口教育资助实现全覆盖；健康扶贫医疗保障政策全面落实，贫困人口住院合规报销比例达90%以上；改造农村危房2.97万户。上海对口帮扶成果丰硕。

　　二、扎实推进乡村振兴，“三农”工作开创新局面。始终把“三农”工作摆在重中之重的位置，深入推进农村产业革命，调减低效作物121万亩，粮经比调整到37：63。农业五大主导产业更加巩固，红粱、食用菌等特色产业蓬勃发展，农业总产值达550亿元，农产品加工业产值460亿元。县级以上农业园区185个，市级以上龙头企业695家，专业合作社7546个，建设集配中心12个，冷库容量突破100万立方米。“三品一标”认证面积达850万亩、产品957个，地理标志农产品12个，遵义绿色优质农产品“走出大山，风行天下”。世界辣椒加工贸易基地建设有序推进，中国辣椒城一期建成营业，辣椒招商走进意大利、俄罗斯，中国遵义辣椒指数面向全球发布。推进村庄“三清一改”，农村人居环境持续改善，整治老旧住房5万户，改建农村户用厕所12.1万户。“大棚房”整治扎实开展，重大疫情有效防控。农民精神文化生活更加丰富，农民丰收节精彩亮相，新时代农民讲习所惠及群众百万人以上。

　　三、深化供给侧结构性改革，产业发展取得新成效。纵深推进工业强市战略，深入实施“双千工程”，编制实施“两计划一规划”，工业总量和增速继续领跑全省，规模工业利润增长20%以上。世界酱香白酒产业基地建设加快推进，“一带两区”白酒产业布局持续巩固，“遵义十大名酒”影响不断扩大。传统产业加快转型升级，完成遵义卷烟厂、华润雪花啤酒、中航电梯异地技改。引进亿元以上项目204个，完成工业投资550亿元，务川氧化铝、绥阳煤电锰等项目投产在即。电子信息、新能源汽车等新兴产业快速成长。两化融合、智能制造等试点示范初见成效。工业转型发展经验获国务院表彰。黔川渝结合部医疗康养中心建设有序推进，遵义康养示范中心、桃花江康养旅游示范基地、赤水医疗康养小镇等项目稳步实施。新增3A级以上景区48个，赤水河谷旅游度假区获“国家级旅游度假区”称号，游客接待量和旅游综合收入分别增长30%以上。“引金入遵”工程扎实推进，泰永长征登陆深圳中小板，新增2家新三板挂牌企业，银河期货遵义营业部开业。深入实施大数据战略行动，完成大数据产业投资80亿元，电子商务交易额增长39%，发展水平居全省第二。国家物流标准化试点城市建设成效显著，48个商贸物流项目完成投资140亿元，高铁新城、黔北物流新城一期迅速推进，快递物流网络体系加快形成。绿地红城项目顺利启动。成功举办茶博会、辣博会、“遵义十大名酒”展销会等展会活动。组建文旅集团，61个文化产业项目加快建设。

　　四、加快公共设施建设，城乡面貌呈现新变化。加快新型城镇化建设，城镇化率达53%。新一轮城市总规编制基本完成，绥阳、湄潭撤县设区和习水撤县设市有序推进。“城市双修”扎实开展，完成长沙路、人民路等6条城区道路改造，新增停车位1.3万个，66个海绵型示范路、示范公园、示范小区、生态景观示范河项目全面启动。房地产市场健康发展，成品住宅建设稳步推进。34个特色小城镇加快建设，实施城镇棚户区改造7.9万套。全国文明城市创建成果持续巩固，启动生活垃圾分类试点，70座垃圾中转站主体完工，44座污水处理厂加快建设，新改建城镇公厕95座，国家卫生城市、卫生县城实现全覆盖，卫生乡镇数量名列全省第一。综合交通枢纽建设扎实推进，新舟机场二期改扩建工程正式启动,旅客吞吐量突破200万人次，成为全省首个跨入中型机场行列的支线机场;茅台机场成为全国首个通航一年旅客吞吐量超百万的支线机场。渝贵铁路建成通车，川黔铁路外迁工程顺利接驳通车，遵泸铁路、城区轨道交通项目前期工作顺利开展。正习、余遵等5条高速加快建设，江习古、务德高速遵义段建成通车。改造提升国省干道260公里，建制村通客运全覆盖。乌江渡、桐梓河等航运工程进展顺利。新开工骨干水源工程13个，10座水库下闸蓄水，治理病险水库12座。绥阳500千伏输变电工程建成运营。行政村4G网络和光纤宽带全覆盖，荣获“宽带中国优秀示范城市”称号。

　　五、全力打好污染防治攻坚战，生态质量实现新提升。深入实施大生态战略行动，全力打好污染防治“五场战役”，国家生态文明建设示范市创建扎实推进。深入开展环保“利剑”行动，聚焦城市黑臭水体、饮用水源保护、企业违规排放、违法建设用地、森林植被破坏等突出问题，中央环保督察反馈问题整改完成率94%。绿色遵义三年行动计划有序开展，完成营造林90万亩，森林覆盖率达59.62%，治理水土流失面积80.2平方公里。积极推进“治污治水·洁净家园”五年攻坚行动，主要河流监测断面水质优良率、县城以上集中式饮用水水质达标率100%，城镇生活污水处理率94.9%，城乡生活垃圾无害化处理率87.9%。大力防治农业面源污染，绿色防控覆盖率30.03%，畜禽粪污综合利用率64%。全面推行河长制，《遵义市湘江保护条例》获省人大常委会批准，重点水域网箱养殖全部取缔。用水总量、用水效率、限制纳污“三条红线”低于省控目标。中心城区环境空气质量优良率98.4%。赤水被生态环境部命名为“绿水青山就是金山银山”实践创新基地，仁怀成功创建国家生态文明建设示范市。

　　六、强力推进改革开放，城市发展增添新动力。“放管服”改革深入实施，取消、下放市级行政许可和非行政许可18项，清理规范中介服务事项50项，行政审批环节压缩23%，审批时限压缩58%。综合行政执法体制、税收征管体制等17项改革任务全面完成，国家农村集体产权制度改革试点、市属国有企业改革重组等67项改革成效明显。商事制度、财政金融、基础教育等改革稳步实施。公立医院综合改革获国务院表彰。庆祝改革开放40周年系列活动隆重举行。遵义综保区封关运行，入驻企业45家。完成外贸进出口总额4亿美元，实际利用外资4.5亿美元。沪遵、渝遵合作更加紧密，先后与绿地、万达等知名企业及泸州、宜宾、南川等地建立战略合作关系，招商引资到位资金增长14.9%。黔川渝结合部教育科创中心建设有序推进，成功列入国家创新型城市，国家高新区、农高区申报取得实质性进展，遵义院士工作中心产学研一体化进程加快。新增国家高新技术企业45家，新建市级以上科技创新平台和人才团队35个,综合科技进步指数达79%。民营经济新增市场主体11万户，实现增加值1600亿元，占地区生产总值的53.3%。

　　七、全力保障和改善民生，社会事业实现新进步。“十件民生实事”累计完成投资329.2亿元。教育事业加快发展，开工建设教育类工程1272个，已建成1156个；义务教育基本均衡全域通过国家验收；国家特殊教育改革试验区建设任务全面完成；遵义医学院更名遵义医科大学，播州区中等职业学校成为国家中职教育改革发展示范学校，中国足球运动学院西南分院建成招生。“百院大战”深入开展，市中医院通过三甲评审，三级医院增至10家、二甲医院增至22家，第九次蝉联全国无偿献血先进城市，城乡医疗卫生机构标准化建设达83%以上。社会保障不断加强，城镇新增就业13.6万人，农村劳动力转移就业14.1万人，城镇登记失业率控制在4.2%以内；进城落户农民工纳入住房公积金政策覆盖范围，城乡三项医保参保率95%以上；最低生活保障、特困人员供养等政策全面落地。全国著名红色文化传承基地建设积极推进，毛主席住居等红色文化综合体加快建设，红色教育培训蓬勃发展，红色文化论坛精彩纷呈。《出山记》《天渠》全国上映，《遵义市志》《遵义丛书》出版发行，荣获“中华诗词之市”称号，遵义大剧院、文化馆、美术馆建成投用，成功承办第九届中国少儿合唱节。精彩承办省十运会，遵义选手获金牌数、奖牌数全省第一，遵义奥体中心、遵义体校新蒲校区、贵州卧龙湖水上运动基地建成投用。持续开展扫黑除恶、打击“两抢一盗”、禁毒人民战争等专项行动，矛盾纠纷和信访遗留问题不断化解，成功处置98个房开突出问题，政府投资项目历史遗留问题有序化解。安全生产实现双降，社会安全指数保持在98%以上。国防动员能力不断加强，兵役登记率达100%。外事侨务、民族宗教、人民防空、粮食安全、气象水文、防灾减灾、档案管理、应急管理、妇女儿童、残疾人、红十字等社会事业全面进步。

　　八、从严加强政府自身建设，工作效能得到新提高。增强“四个意识”，坚定“四个自信”，做到“两个维护”，深入开展贯彻落实习近平总书记重要指示批示精神“回头看”，全面抓好中央和省委巡视、国务院大督查反馈意见整改落实。围绕“解放思想敢担当，真抓实干有作为”主题教育活动，推动政府效能不断提高。修订完善《政府工作规则》《政府会议制度》，自觉接受人大及其常委会法律监督和工作监督、政协民主监督、审计监督、新闻媒体和群众监督，办理人大代表建议172件、政协委员提案358件。落实政府法律顾问制度，行政应诉、行政复议等步入规范化、常态化轨道。开展营商环境整治百日攻坚行动，33个业务系统迁入“云上贵州”平台运行。深入开展政府系统党风廉政建设和反腐败工作，全面落实党风廉政建设主体责任和“一岗双责”，政风行风持续好转。

　　各位代表，这些成绩的取得，得益于党中央、国务院和省委、省政府亲切关怀，得益于市委坚强领导，得益于市人大、市政协监督支持，得益于全市干部群众艰苦奋斗，得益于社会各界倾力相助和上海对口帮扶。在此，我谨代表市人民政府，向全市各族人民、知识分子、广大干部职工，向各民主党派、工商联、人民团体、无党派人士，向驻遵解放军指战员、民兵预备役人员、武警官兵、公安干警和消防救援队伍指战员，向历届老领导老同志和所有关心支持遵义经济社会发展的各界人士，表示衷心感谢并致以崇高敬意！

　　在充分肯定成绩的同时，我们也清醒看到稳增长的压力持续加大，各类风险交织迭加，特别是部分地方债务负担较重，部分企业、项目融资受阻，新开工项目个数和投资出现下降，企业生产经营困难。同时，也有我们对困难估计不足，创造性抓工作有差距，导致地区生产总值、投资、工业、消费等预期增长目标未能全面实现。经济社会发展仍然面临不少挑战和问题，主要是：发展不平衡不充分问题依然突出，拥有的资源禀赋和比较优势没有充分发挥，发展质量和效益仍然不高；新旧动能转换较慢，产业结构仍需优化；政府投资比重较大，产业投资占比偏低，社会投资活力不足；“三大攻坚战”任务依然艰巨；民生领域仍有欠账；营商环境还需改善，少数干部不担当不作为慢作为乱作为现象时有发生。针对这些问题，我们将认真加以解决。

　　2019年的总体要求、奋斗目标及重点任务

　　今年是新中国成立70周年，是决胜全面建成小康社会第一个百年奋斗目标的关键之年，是我市脱贫攻坚决战之年，做好全年经济社会发展各项工作至关重要、意义重大。我们既要看到经济下行压力加大、投资增长乏力、化解债务风险任务艰巨等严峻挑战；又要看到中央决战脱贫攻坚、做强实体经济、推动乡村振兴、新时代实施西部大开发战略政策效应持续迭加释放；省委省政府持之以恒推进三大战略行动，加快三大国家级试验区建设的战略机遇；特别是市委五届五次、六次全会作出建设黔川渝结合部中心城市、推动工业高质量发展等一系列重大战略部署，为我们补短板强弱项、调结构促转型，加快经济社会发展注入了强劲动力；同时，遵义经济外向度不高，经济社会发展稳中向好的基本面没有变。稳增长，关键在稳信心，核心在善作为，基础在抓项目。我们必须牢牢把握遵义经济发展的时代特征，辩证看待和正确处理好政府与市场、扩大投资与防范债务风险、工业化与城镇化、经济发展与环境保护四个关系，审时度势，保持定力，精准发力，推动遵义经济社会持续健康发展。

　　今年政府工作的总体要求是：以习近平新时代中国特色社会主义思想为指导，深入贯彻党的十九大及十九届二中、三中全会精神和习近平总书记在贵州省代表团重要讲话精神，统筹推进“五位一体”总体布局，协调推进“四个全面”战略布局，坚持稳中求进工作总基调，坚持新发展理念，贯彻落实“巩固、增强、提升、畅通”八字方针，以供给侧结构性改革为主线，深化市场化改革，扩大高水平开放，推动经济高质量发展，遵循“六稳”要求，坚决打好三大攻坚战，实施三大战略行动，守牢发展和生态两条底线，全面落实市委五届五次、六次全会决策部署，全力抓好稳增长、促改革、调结构、惠民生、防风险各项工作，提高人民群众获得感、幸福感、安全感，为全面建成小康社会收官打下决定性基础，以优异的成绩庆祝新中国成立70周年！

　　今年的主要预期目标是：地区生产总值增长11%左右；规模工业增加值增长11%；固定资产投资增长18%；社会消费品零售总额增长11.5%；一般公共预算收入增长7%；城乡居民人均可支配收入分别增长9%、10%；金融机构存贷款余额分别增长5%、18%；全面小康实现程度93.5%；居民消费价格指数、节能减排降碳等指标控制在省下达的目标范围内。

　　围绕上述目标，主要抓好以下工作任务：

　　一、决战决胜脱贫攻坚，夯实全面小康基础。坚决落实精准方略。对标“一达标两不愁三保障”，聚焦深度贫困，奋力夺取决战之年的根本性胜利。减少贫困人口6万人以上，贫困发生率下降到1%以内，实现45个贫困村出列，务川、道真脱贫摘帽，正安达到国家贫困县退出评估标准。持续减少面上“插花型”贫困现状，巩固提升已脱贫摘帽县（市）脱贫成果。持续打好四场硬仗。用好用足绿色产业发展扶贫基金，因地制宜培育壮大主导产业和特色产业，巩固提升贫困人口产业覆盖水平；新增扶贫龙头企业20家以上，发展订单农业300万亩。确保易地扶贫搬迁入住2.36万人，完善225个安置点后续配套服务，确保贫困群众“搬得出、稳得住、能致富”。新建和改造农村电网1.03万公里，持续提升农村4G网络通信质量。保障10万名农村学前儿童、55万名农村义务教育学生营养改善计划实施，完成2.17万贫困人口职业技能免费培训；确保建档立卡贫困人口全部参加新农合，住院合规报销比例保持在90%以上，贫困村和安置点卫生室全部实现规范化建设。完善扶贫工作体系。巩固提升产业发展“一长两短”和利益联结、易地搬迁“三大体系”、医疗扶贫“四精准一便民”和全员育人导师制等工作机制，持续保障政策精准滴灌。深化东西部扶贫协作，推进沪遵深度合作，推动上海对口帮扶与深度贫困村、贫困县中小学和医院实现结对帮扶全覆盖。抓好干部驻村、结对帮扶、千企帮村等工作。致力扶志扶智，强化“文军扶贫”“法治扶贫”，增强群众脱贫内生动力和自我发展能力，防止脱贫人口返贫。深入开展“城市解困·和弦行动”。

　　二、实施乡村振兴战略，推进美丽乡村建设。推动农业高质量发展。加快发展现代山地特色高效农业，粮食产量稳定在235万吨以上，农业增加值增长6.8%。大力实施农村产业革命，新增土地流转100万亩，以500亩以上坝区为突破口，不断优化产业结构和布局，实现茶叶、辣椒、竹、生态畜禽综合产值分别达200亿元以上，中药材综合产值100亿元以上；推进红粱、花椒等特色产业发展，种植红粱100万亩，新增花椒20万亩。巩固提升农业园区建设水平和质量，大力培育新型农业经营主体，组建遵义茶业集团、遵义辣椒集团，壮大遵义供销集团，市级以上龙头企业增至745家，农民专业合作社增至7800个，实现农产品加工业总产值500亿元。推进遵义红、遵义绿、遵义朝天椒等品牌发展壮大，“三品一标”认证面积增至900万亩以上、产品1000个以上，地理标志农产品16个。强力推进世界辣椒加工贸易基地建设，实施辣椒“种源工程”12万亩以上，加快中国辣椒城升级改造，实现干辣椒交易40万吨，常态发布中国遵义辣椒指数，加快实现“中国辣椒、遵义定价、全球销售”。深化农村综合改革。积极推广湄潭“四确五定”和播州“五权五定”经验，完成农村集体产权制度改革试点任务。深化农村“三变”改革,推动农村“三社融合”，培育农村发展新动能，支持各类人才返乡下乡创新创业，拓展农村就业空间和农民增收渠道。持续推进美丽乡村建设。深入实施“四在农家·美丽乡村”小康升级行动计划，加强农村人居环境整治，推进农村“三改”。持续推进“治污治水·洁净家园”五年攻坚行动，完善农村垃圾处置机制，提升农村污水处理设施建管运能力。持续推进“大棚房”整治和重大疫情防控。探索以乡镇为主的基层社会治理创新模式，加强农村基层基础工作，健全自治、法治、德治相结合的乡村治理体系，推动网格化实体运行，提升乡村治理水平。新办2000个新时代农民讲习所，开展“明礼知耻·崇德向善”道德大讲堂活动，提升乡村文明水平。

　　三、构建现代产业体系，加快新旧动能转换。推动工业高质量发展。深入实施工业强市战略，聚力发展“七大主导产业”，奋力建设西部现代工业强市。加快打造世界酱香白酒产业基地，推动白酒企业兼并重组，培育壮大一批新型白酒企业，提升“遵义十大名酒”影响力；支持茅台集团加快形成年产5万千升茅台酒、10万千升系列酒生产规模，重点推动茅台酒厂中华片区和201厂技改、国台酒业万吨技改等项目，全力打造“中国酱香白酒产区”地理标志和“中国酱香·赤水河谷”地域品牌。深入开展“千企改造”，推进装备制造、能源、材料等传统产业转型升级，加快贵州钢绳、遵义钛厂等企业异地技改。积极推动遵义卷烟厂扩大高档烟比例。加快提升煤矿开采智能化机械化程度，提高煤矿产能。夯实工业园区平台，实施“千企引进”战略，围绕主导产业和本地资源培育龙头，立足补链、强链、延链开展招商引资，引进产业项目330个，开工建设亿元以上工业项目100个。加快国家军民融合创新试验区建设，强化与航天科工集团合作，振兴军民融合产业。推进两化融合，着力打造一批“数字车间”“智慧工厂”。积极发展清洁能源，确保正安页岩气开采利用取得实质性突破。大力发展航空航天、新能源、新材料、节能环保、高端装备制造等战略性新兴产业,支持巴斯巴、凯山特种车等新能源汽车发展。不断提升三产发展水平。加快“三区三中心”建设，引领高端产业集聚发展，推动第三产业量质齐升，有效扩大居民消费。加快黔川渝结合部医疗康养中心建设，推进医养游融合发展，打造“醉美遵义·康养福地”。建成遵义康养示范中心，加快赤水河谷国家级旅游度假区、桃花江康养旅游示范基地建设，启动播州恒大养生谷、桐梓康养旅游度假区、洛安江生态康养小镇、金鼎山特色小镇等项目建设。持续推进“创A工程”，新增3A级以上景区42个，启动湄潭茶壶景区提升工程，加快推进海龙屯、云门囤等景区建设，加强旅游市场营销管理，实现游客接待量、旅游综合收入分别增长20%、25%以上，争取国家全域旅游示范区创建通过首批验收。持续优化金融业发展环境，提升金融服务实体经济水平，引进金融企业3家，培育一批中小企业在新三板挂牌。深入实施大数据战略行动，加快“数字经济”建设，启动实施大数据新领域百企引领行动，培育一批软件和信息技术服务企业，促进物联网产业应用发展。强化商贸物流、会展、文化等产业发展，加快构建“一园区两中转四层级”快递物流体系，推动黔北物流新城等项目建设，建成启用黔北快递物流园；引进一批会展文化企业，加快绿地红城会展中心、乌江古村落等会展文化项目建设，办好茶博会、辣博会、省第五届大健康产业发展大会、国际盆景赏石大会等展会活动。大力推动民营经济发展。全面贯彻民营经济“两个毫不动摇”“三个没有变”的总方针，扎实开展民营经济政策落实、金融服务、降本减负、扩大民间投资、营商环境整治、领导干部联系服务企业“六大专项行动”，出台支持民营经济发展、农民工返乡创业的政策措施，强化政策落实，提升服务质量，依法保护民营经济主体合法权益。新增市场主体6万户以上，完成民营经济增加值1800亿元，占地区生产总值比重54%以上。

　　四、建设新型城镇体系，推动城镇品质提升。加快推进新型城镇建设。围绕构建500万人口中心城市框架，提升城乡建设发展水平，城镇化率提高到54%以上。建设中心城区“控规一张图”管理平台，完善“两图合一”。推进城市基础设施、公共服务设施等专项规划编制。力争绥阳、湄潭撤县设区，加快推进习水撤县设市。启动遵义至绥阳、湄潭、桐梓、仁怀快速通道建设。加快武汉路、新南快线、航新快线等骨干道路建设，确保凤新快线建成通车。启动新火车站东广场等重点项目建设。加快实施“城市双修”，推进老城区路网改造和居住设施配套能力建设，完成海绵城市项目14个、城镇一类公厕37座。实施“增绿添色”工程，建成一批城市公园、示范小区、生态景观项目。因地制宜推进播州泮水、仁怀九仓、中国习酒城等30个特色小城镇建设。全面提升城市管理水平。持续巩固全国文明城市创建成果，强化市容环境整治，从严打击“两违”行为。鼓励建设立体车库，中心城区新增停车位5000个。加快城乡垃圾收运处置设施建设，新建垃圾中转站18个，中心城区推行生活垃圾强制分类和处置监管，启动建设东部城区垃圾焚烧发电工程。持续巩固国家卫生城镇创建成果，创建国家卫生乡镇5个。强化房地产市场监管，促进房地产市场平稳健康发展。

　　五、强化基础设施建设，不断改善发展条件。加快建设黔川渝结合部综合交通枢纽。完成交通投资240亿元。加强航空运输能力建设，新舟机场和茅台机场旅客吞吐量实现360万人次以上，加快新舟机场改扩建工程建设，完成赤水、播州、正安通用机场前期工作。加快昭黔、遵泸及古蔺大村至遵义铁路前期工作，启动瓮马铁路延伸线、遵义动车所和城市轨道交通1号线建设。开工建设金仁桐、桐新高速公路，加快余遵、湄石、遵仁、重遵扩容等高速公路建设，确保绥正、正习、乐冷高速公路建成通车，总里程增至1321公里。改造干线公路180公里。加大农村公路管护力度。启动余庆河、芙蓉江等库区航运工程建设。不断完善城乡水利设施。完成水利投资50亿元。新开工观音水库等骨干水源工程10个以上，确保6座水库下闸蓄水。治理病险水库23座。开工建设中小河流及大江大河治理项目11个。健全水利设施管理机制。提升电力通信承载能力。完成投资17.3亿元，建成播州500千伏、绥阳220千伏输变电工程，开工建设习水500千伏和正安、习水220千伏输变电工程。开展“数字设施”提升行动，完成投资10亿元以上，推进全光网城市建设，通信光缆达17.3万公里，省网骨干带宽达2500G，30户以上自然村4G网络覆盖率达95%，积极推进物联网基站和5G试验网建设。

　　六、狠抓生态文明建设，不断筑牢生态底线。推动绿色发展。强化节能减排，加强煤化工、火电厂、水泥厂等重点企业“三废”监控，确保单位产品能耗控制在国家和省限额标准以内。推进国家节水型城市创建，用水总量、万元GDP和工业增加值用水量控制在目标值内。单位GDP建设用地下降4.7%。强化资源循环利用，提高粉煤灰、酒糟等大宗工业固废物综合利用率。加强生态建设。加快国家生态文明建设示范市、县创建。抓好裸露山体绿化，实施营造林90万亩，森林覆盖率提高到60%以上。严格保护耕地和基本农田，确保耕地保有量不低于1206万亩，基本农田保护面积不低于1027万亩。治理水土流失面积60平方公里。强化环境治理。坚持铁腕治污，坚决打好蓝天、碧水、净土保卫战，全面整改落实中央环保督察问题。落实河长制，强化重点水域综合治理保护，确保监测断面水质优良率达100%，县城以上集中式饮用水水质全部达标，中心城区环境空气质量优良率持续巩固。推进城乡生活污水和垃圾处理设施建设，实现城镇污水处理率95%，中心城区和县城生活垃圾无害化处理率分别达95%、90%。全面推广集中连片村寨一体化生态整治，强化农业面源污染治理，持续减少化肥农药用量，绿色防控覆盖率达32%以上，畜禽粪污综合利用率达68%。抓好第二次全国污染源普查工作。

　　七、深化改革开放创新，全面增强发展活力。抓好改革关键一招。深入推进供给侧结构性改革，以“改革指数”提升“发展指数”。完成市县两级政府机构改革，建立健全科学管理机制，推动机构高效运转。深化“放管服”改革，扎实推进行政审批“六个一”、商事制度等改革事项。不断优化营商环境，将企业开办时间压缩到3个工作日以内，工程建设项目审批时间压缩到80个工作日以内，不动产登记办理时间压缩到5个工作日以内。持续深化市属国有企业改革，推动实体化、市场化转型。完成农村信用社改制工作。全面推进基础教育、医疗卫生、生态文明、县级供销社等领域改革。纵深推进开放合作。积极搭乘渝新欧班列和南向通道快车，扩大与“一带一路”沿线国家和长江经济带沿线城市交流合作。建立完善黔川渝结合部城市间协作机制，持续深化沪遵、渝遵合作。加快新舟机场正式航空口岸建设。完善国开区、综保区等开放平台功能。深入开展产业大招商，完善考核机制，招商引资到位资金增长14%。确保外贸进出口总额增长10%，实际利用外资增长10%。加快建设黔川渝结合部教育科创中心。深入实施教育科创中心三年行动计划，全力打造西部非省会教育强市，提升国家创新型城市水平，推进国家高新区、农高区建设。与北京师范大学合作建设遵义研究院，深化与华东师范大学等高校合作，建设一批带动力强的科技创新平台。出台促进科技创新的政策措施，培育国家高新技术企业和省级科技型企业60家以上、科技服务机构10个以上，引进培育一批领军人才及创新团队，加快构建产学研深度融合的科技创新体系。大力推动“大众创业、万众创新”，完善创新创业孵化基地功能，重点扶持大学生创新创业。全社会研发经费占地区生产总值比重0.6%，综合科技进步指数82%以上。

　　八、着力重大风险防控，确保社会和谐稳定。全力防控政府债务风险。按照坚定、可控、有序、适度的要求，健全债务风险防控机制，落实债务主体责任，坚决遏制债务增量、减少债务存量。通过盘活资源、资金、资产，积极申请发行政府专项债券，推进PPP项目建设模式，重组、审减、处置、整合、停建、转股和预算偿还一批政府债务，确保不发生区域性、系统性金融风险。全力防控社会稳定风险。强化基层治理能力建设，纵深推进扫黑除恶专项斗争，为人民群众营造和谐稳定的生活环境。完善禁毒“五大体系”，打赢新时代禁毒人民战争。开展“七五”普法，全面推广新时代“枫桥经验”，推进“雪亮工程”建设，发挥综合治理“五治”作用。完善多元化解矛盾纠纷问题机制，推动信访积案化解。全力防控安全生产风险。强化安全生产底线思维和红线意识，落实安全生产“党政同责、一岗双责、齐抓共管、失职追责”机制。加强重点行业领域安全生产隐患专项排查整治，从源头遏制安全生产事故发生，确保安全生产形势稳定向好。

　　九、推进民生事业发展，切实增进人民福祉。促进就业创业。深入开展职业技能培训，重点保障易地搬迁群众、返乡农民工、高校毕业生、城镇困难人员、退役军人等创业就业，城镇新增就业12.6万人，转移农村劳动力就业10.5万人，城镇登记失业率控制在4.2%以内。坚持教育优先发展。推进中心城区基础教育发展提升三年行动计划，公办幼儿园比例提高到47%；巩固提升义务教育基本均衡创建成果，新改扩建中心城区学校21所、新增学位3.6万个，基本消除大班额。引导高中阶段教育高效发展，推动高职高专优质内涵发展，支持驻遵高校积极争取博士、硕士学位授予单位和授权点资格。稳步实施第二期特殊教育行动计划。积极支持社会力量办学。加快健康遵义建设。深入实施“百院大战”，创建三甲医院2家，建成市妇女儿童医院、市中医院新蒲院区，遵义医科大二附院、市公共卫生中心。完善和巩固医疗卫生机构标准化建设，推进全面分级诊疗，加快医联体医共体建设。深入开展生育健康全程服务，完善全面两孩配套政策。鼓励和规范社会办医。推进全民健身和竞技体育发展。着力红色文化引领。围绕加快建设全国著名红色文化传承基地，组建遵义红色文化专家团队，强化红色文化资源保护利用，加快播州长征园等红色文化项目建设，抓好全国红色旅游五好讲解员建设行动试点，申报挂牌一批爱国主义教育示范基地，推出红色经典影视作品，开发红色精品课程，形成有影响力的红色文化研究成果，提升遵义红色文化影响力。巩固提升“五大群众文化活动品牌”，组织申报第八批全国重点文物保护单位，巩固“中华诗词之市”创建成果。提升文化体育场馆运营服务水平。全面加强社会保障。实施“全民参保计划”，提升高风险行业工伤保险参保率，推进城乡居民医保并轨，将住房公积金制度覆盖到城市灵活就业人员。持续推进劳动保障大数据监测分析试点工作。统筹推进城乡社会救助体系建设，加强农村“三留守”人员、城镇困难职工、失独失能老人关爱保护。构建社区居家养老、社会服务养老和农村敬老院结合的城乡养老服务体系。完善殡葬基本公共服务制度，规划建设汇川、新蒲殡仪馆。争创全国民族团结进步示范市，确保蝉联全国双拥模范城市。深化国防动员备战准备，推动国防动员深度发展。推动外事侨务、人民防空、气象水文、疾病防控、应急管理、粮食安全、妇女儿童、档案管理、地方志、老年人、残疾人、红十字等社会事业再上新台阶。

　　全力办好十大民生实事。一是完成80个全面改薄项目，新改扩建镇村幼儿园100所。二是建成专业儿童医院1所，建成智慧卫生急救站100个。三是开展就业困难群体职业技能培训3万人、实现就业2万人。四是持续提高城乡低保标准，城乡居民基本养老金提高24%。五是新建改造农村户用卫生厕所1.2万户、农村公厕590座、旅游公厕100座。六是城镇棚户区改造基本建成3.59万户，建成公租房1774套。七是完成播州区公交同城化、国有化改造。八是改扩建遵义、务川精神病院，建成40个日间照料中心。九是农村饮水安全巩固提升工程解决受益人口4.55万人。十是设立12345市民服务热线，建成政务服务“一张网”云平台，实现政务服务“一门进，并联审，限时办，一窗结”。

　　十、加强政府自身建设，全面提升工作效能。旗帜鲜明讲政治。牢固树立“四个意识”，坚定“四个自信”，做到“四个服从”，坚决维护习近平总书记核心地位，坚决维护党中央权威和集中统一领导，坚决服从省委、省政府和市委领导，全面推动决策部署落实，用工作实绩把对党的绝对忠诚镌刻在黔北大地上。真抓实干强担当。全面增强推动经济社会高质量发展的工作本领，大力弘扬求真务实、真抓实干的作风，敢于克难攻坚，敢于动真碰硬，完善督考合一、正向激励和容错机制，激励广大干部主动作为、担当尽责，做到政府决策的每项工作都能落实，政府承诺的每件事都能兑现，干出经得起历史和人民检验的工作业绩。依法行政重规范。牢固树立法治意识和法治思维，严格依法行政，认真执行风险评估、合法性审查、行政复议、政府法律顾问、行政应诉等制度，自觉接受人大法律监督、工作监督和政协民主监督，以及群众监督、审计监督、舆论监督，习惯在约束中干事、在监督下履职，确保政府工作在法治的轨道上、人民的监督下规范运行。廉洁自律守规矩。全面落实党风廉政建设责任制，时刻把纪律和规矩挺在前面，模范遵守中央、省委和市委作风建设各项规定，严格“三公”经费管理，强化财政资金使用效果评估考核，坚定不移兴“三风”、纠“四风”、树新风。坚决防止靠山吃山、假公济私、以权谋私等腐败问题发生，确保政府系统风清气正。

　　各位代表！奋斗创造幸福，实干成就梦想。让我们更加紧密团结在以习近平同志为核心的党中央周围，在省委、省政府和市委的坚强领导下，牢记嘱托、感恩奋进，以新视野把握新形势，以新观念开创新局面，以新举措推动新发展，为加快建设黔川渝结合部中心城市而不懈奋斗！ 0
